# From cell membrane to the nucleus: an emerging role of E-cadherin in gene transcriptional regulation

**DOI:** 10.1111/jcmm.12340

**Published:** 2014-08-28

**Authors:** Wenjun Du, Xi Liu, Guiling Fan, Xingsheng Zhao, Yanying Sun, Tianzhen Wang, Ran Zhao, Guangyu Wang, Ci Zhao, Yuanyuan Zhu, Fei Ye, Xiaoming Jin, Fengmin Zhang, Zhaohua Zhong, Xiaobo Li

**Affiliations:** a Department of Digestion, Shandong Provincial Qianfoshan Hospital, Shandong UniversityJinan, Shandong Province, China; b Department of Cardiovascular, Inner Mongolia People's HospitalHohhot, Inner Mongolia, China; c Department of Clinical Etiology, Qiqihar Medical UniversityQiqihar, Heilongjiang Province, China; d Department of Pathology, Harbin Medical UniversityHarbin, Heilongjiang Province, China; e Department of Gastrointestinal Medical Oncology, The Affiliated Tumor Hospital of Harbin Medical UniversityHarbin, Heilongjiang, China; f Department of Microbiology, Harbin Medical UniversityHarbin, Heilongjiang Province, China; g Center of Translational Medicine, Harbin Medical UniversityHarbin, Heilongjiang Province, China

**Keywords:** E-cadherin, cell signalling, gene expression

## Abstract

E-cadherin is a well-known mediator of cell–cell adherens junctions. However, many other functions of E-cadherin have been reported. Collectively, the available data suggest that E-cadherin may also act as a gene transcriptional regulator. Here, evidence supporting this claim is reviewed, and possible mechanisms of action are discussed. E-cadherin has been shown to modulate the activity of several notable cell signalling pathways, and given that most of these pathways in turn regulate gene expression, we proposed that E-cadherin may regulate gene transcription by affecting these pathways. Additionally, E-cadherin has been shown to accumulate in the nucleus where documentation of an E-cadherin fragment bound to DNA suggests that E-cadherin may directly regulate gene transcription. In summary, from the cell membrane to the nucleus, a role for E-cadherin in gene transcription may be emerging. Studies specifically focused on this potential role would allow for a more thorough understanding of this transmembrane glycoprotein in mediating intra- and intercellular activities.

## An emerging function of E-cadherin: gene transcriptional regulation

E-cadherin is a member of the cadherin family, a family of transmembrane glycoproteins responsible for calcium-dependent cell adhesion that are the key structural components of adherens junctions (AJs) [1]. E-cadherin is present in epithelial tissues and composed of a single-pass transmembrane region, a cytoplasmic region and an ectoregion [1].

Recently, reports have described other functions of E-cadherin beyond its role in mediating AJs. In particular, E-cadherin was found to regulate gene expression. Sasaki *et al*. first reported that a loss of E-cadherin in invasive breast cancer cells resulted in an increase of Bcl-2 expression, contributing to chemotherapy resistance in tumour cells [2]. Wang *et al*. found an inverse correlation between E-cadherin and epidermal growth factor (EGF) receptor (EGFR) expression in tissue specimens of head and neck sarcoma, further demonstrating that EGFR signalling activation inhibited the expression of E-cadherin, and knockdown of E-cadherin resulted in the elevation of EGFR transcription [3]. Strumane *et al*. found an inverse correlation between E-cadherin and human Nanos1 expression in various cell lines and showed that the re-expression of E-cadherin in a human breast cancer cell line decreased hNanos1 expression [4]. The first systematic study on the role of E-cadherin in global gene transcription was performed by Onder *et al*. in 2008. They inhibited the function of E-cadherin through either siRNA-mediated knockdown or expression of a truncated form of E-cadherin (cytoplasmic-region) in human mammary epithelial cells and found that the expression of many genes had been altered significantly as a result. Indeed, they showed that it was the loss of E-cadherin itself and not the loss of cell–cell contacts or the subsequent activation of β-catenin that contributed mostly to this alteration. This was the first time to show that a loss of E-cadherin resulted in the transcriptional elevation of Twist and ZEB1, two well-known transcriptional repressors of E-cadherin [5]. Recently, Francesca *et al*. compared global transcript expression in E-cadherin-null (E-cad-/-) embryonic stem (ES) cells and E-cadherin wide-type ES cells, showing that E-cadherin depletion led to the altered expression of 2265 genes. Notably, they did not detect an elevation of β-catenin activity after E-cadherin depletion in their model. However, they did observe an enhancement of FGF signalling activity as a result of the increase of FGF5 transcription in E-cad-/- ES cells [6]. These results implied that E-cadherin was a novel regulator of gene transcription, even though the molecular mechanisms involved had not yet been fully detailed. Analysis of the available E-cadherin data, particularly in regard to its regulation of cell signalling pathways, may help shed some light on this issue.

## Mechanism of action: regulation of cell signalling by E-cadherin

Recently, it was reported that E-cadherin was tightly linked to several major signalling pathways, including Wnt/β-catenin, NF-κB, receptor tyrosine kinase (RTK) and GTPase signalling pathways. In most cases, the end-point of the cell signalling pathways is to regulate gene expression and ultimately modulate cellular behaviour. Thus, E-cadherin may regulate gene expression through influencing the transduction of these signals to the nucleus.

### Effects of E-cadherin on the Wnt/β-catenin signalling pathway

**Fig. 1:**
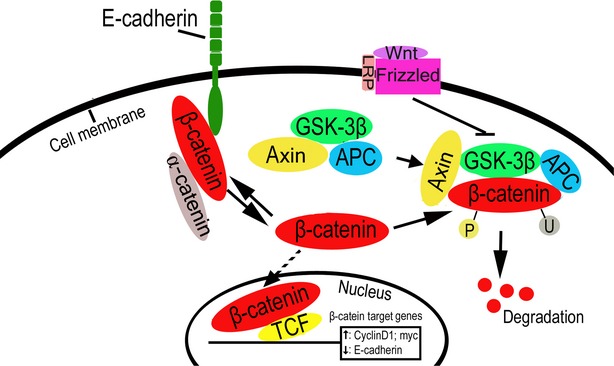
E-cadherin inhibits Wnt/β-catenin signalling. β-catenin can be located in the membrane, cytoplasm or nucleus depending on the status of Wnt signals and the expression and distribution of E-cadherin.

E-cadherin-based AJs share a key component with the Wnt/β-catenin signalling pathway – β-catenin. β-catenin can be found in the membrane, cytoplasm or nucleus depending on the status of Wnt signals and the expression and distribution of E-cadherin (Fig. 1). In normal epithelial cells, β-catenin interacts with and binds to the cytoplasmic tail of E-cadherin and is sequestered at the membrane [7]. When Wnt signals are absent, free β-catenin forms a complex with GSK3β, APC and Axin in the cytoplasm, and is phosphorylated by CK1 and GSK3β. Phosphorylated β-catenin is subsequently degraded through the ubiquitination-proteasome degradation system. While Wnt signalling is active because of Wnt ligand binding to Frizzled receptor, however, GSK3β is displaced from the regulator APC/Axin/GSK3β complex and thus its activity is inhibited, thereby liberating β-catenin, allowing it to accumulate in the cytoplasm and translocate to the nucleus where it can then regulate target gene transcription through an interaction with TCF/LEF family transcription factors and Legless family docking proteins [8].

Over the past few decades, some reporters have surmised that a loss of E-cadherin may elevate the activity of β-catenin, having evaluated its activity through luciferase reporter systems and determination of TCF/β-catenin target gene expression [5,9]. However, it was commonly accepted that E-cadherin loss alone was not sufficient to activate β-catenin signalling [3,6,10–12], requiring instead the presence of other effectors, such as Wnt and FGFR signalling activity [13,14]. With the combined use of time-lapse microscopy and image analysis, the cadherin-bound pool of β-catenin was shown to accumulate at the perinuclear endocytic recycling compartment (ERC) upon AJ dissociation and then translocate into the nucleus upon Wnt signalling pathway activation, which suggests that the ERC may be a site of residence for β-catenin following its liberation from the membrane cadherin complex and prior to entering the nucleus [15]. In most cases, restoration or overexpression of E-cadherin inhibited β-catenin activity by sequestering cytoplasmic β-catenin [9,16–21]. Notably, overexpression of the cytoplasmic region of E-cadherin was sufficient to achieve this response [9,18,21]. These results imply that E-cadherin may be a negative regulator of the Wnt/β-catenin signalling pathway. However, Howard *et al*. recently showed that the ability to bind E-cadherin was necessary for β-catenin's transcriptional activity, and E-cadherin was required for augmented activation of the Wnt/β-catenin pathway *in vivo*, which suggests that E-cadherin could be a positive regulator of the Wnt/β-catenin pathway in certain models [22]. In yet another study, the Wnt/β-catenin pathway seemed to regulate E-cadherin expression. The E-cadherin gene promoter contains TCF/β-catenin binding sites, and Wnt signalling activation represses the expression of E-cadherin in a TCF/β-catenin-dependent manner, which suggests that a feedback circuit may exist between E-cadherin and Wnt/β-catenin signalling [23].

### Effects of E-cadherin on RTK signalling pathways

Growth factors, such as EGF, FGF, TGF and HGF, are known to promote cell proliferation and prevent apoptosis through binding to their receptors in the cell membrane, inducing dimerization of the receptors and concomitant activation of the intracellular tyrosine kinase domains. The activated RTKs can then phosphorylate their substrates, resulting in the activation of multiple downstream signalling pathways, including MAPK (mitogen-activated protein kinase), PI3K/AKT and STAT signalling pathways [24].

As early as 1994, the E-cadherin–β-catenin complex was shown to interact with Erb-B2, a member of the EGF receptor family of RTKs, in the cancer cell membrane [25]. Soon after, several other groups demonstrated that E-cadherin could bind the EGFR [26–28]. In one study, interaction of the extracellular domain of E-cadherin with EGFR was required for the transient activation of EGFR signalling in mammary cells [26]. In another, Pece and Gutkind showed that E-cadherin interacted with EGFR and activated EGFR-mediated MAPK signalling in a ligand-independent manner [28]. More recently, the extracellular domain of soluble E-cadherin was shown to interact with EGFR and activate EGFR-mediated PI3K/AKT and ERK1/2 signalling in breast cancer cells and squamous cell carcinoma [29,30].

**Fig. 2:**
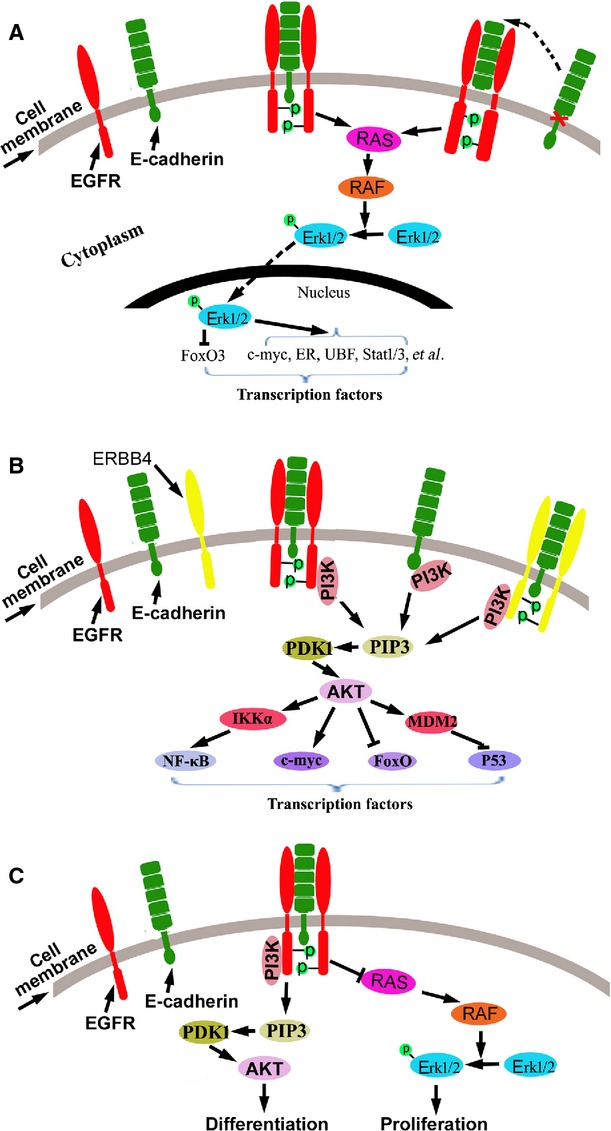
Effects of E-cadherin on RTK signalling. (**A**) E-cadherin or the soluble E-cadherin interacts with EGFR and activates MAPK signalling pathway in cancer cells; (**B**) E-cadherin interacts with EGFR or ERBB4 and activates PI3K/AKT signalling pathway in cancer cells; (**C**) In normal human urothelial cells, E-cadherin inhibited EGFR-mediated MAPK signalling and activated PI3K/AKT signalling.

E-cadherin has also been shown to inhibit EGFR signalling in some experimental contexts. Qian *et al*. demonstrated that E-cadherin could bind EGFR and inhibit the ligation-dependent activation of EGFR signalling in breast cancer and melanoma cells [27].By using microsphere-embedded recombinant E-cadherin protein to form homophilic bonds with E-cadherin at the cell surface, Perrais *et al*. showed that E-cadherin directly transduced growth-inhibitory signals and that E-cadherin ligation inhibited EGFR-mediated transphosphorylation and activation of STAT5 [31]. In NCI-H292 cell lines, E-cadherin was demonstrated to activate EGFR-mediated cell differentiation, but inhibit EGFR-mediated cell proliferation [32]. In normal human urothelial cells, E-cadherin inhibited EGFR-mediated MAPK signalling and activated PI3K/AKT signalling [33]. However, direct binding may not be the only in which EGFR-mediated signalling is modulated by E-cadherin. In fact, knockdown of E-cadherin in head and neck tumour cells was shown to elevate EGFR transcription [3]. Notably, EGFR signalling has also been found to regulate E-cadherin expression and function in tumour cells through inhibiting its transcription and promoting its cleavage, degradation and endocytosis [34–39], suggesting a feedback regulation between E-cadherin and EGFR signalling. Taken together, these results suggest that the regulation of EGFR signalling by E-cadherin is indeed complex (Fig. 2).

In addition to EGFR, E-cadherin is also shown to interact with FGFR. In MCF-7 breast cancer cells, treatment with FGF induced the endocytosis of E-cadherin and FGFR. The interaction of E-cadherin with FGFR was required for the nuclear translocation of FGFR and subsequent activation of FGF-induced MAPK signalling. Overexpression of E-cadherin blocked the endocytosis of both molecules, the nuclear translocation of FGFR and the activation of FGFR-mediated MAPK signalling [40]. In Ewing tumour cells, under anchorage-independent growth conditions, E-cadherin was up-regulated and correlated with the formation of multicellular spheroids and the suppression of anoikis. The mechanism study showed that E-cadherin activated the Erb-B4 RTK coupled with the activation of PI3K/AKT signalling [41].

E-cadherin may also directly regulate PI3K activity. Indeed, PI3K was recruited to the site of cell–cell contact by the ligation of homophilic E-cadherin, resulting in the activation of PI3K signalling [42,43]. Recently, the p85 subunit of PI3K was shown to be directly targeted by the E-cadherin complex and activated in ovarian cancer cells [44].

### Effects of E-cadherin on the GTPase signalling pathways

GTPases are molecular switches that control multiple processes in eukaryotic cells while cycling between a GTP-bound active state and a GDP-bound inactive state. GTPases consist of five major groups: Rho, Ras, Rab, Ran and Arf. Rho GTPases are primarily known for regulating the actin cytoskeleton and cell polarity [45]. Recently, Rho GTPases were also found to regulate gene transcription. For example, in mid-G1 phase of the cell cycle, Rho GTPases inhibited the expression of cyclin/CDK inhibitor P21, but induced the expression of cyclin D1 through promoting the sustained activation of MAPK signalling [46,47]. E-cadherin was found to regulate the activity of Rho, Rac and Cdc42, the three most well-characterized members of the Rho GTPases, implying that E-cadherin may regulate transcription through regulating GTPase signalling activity.

Rac activation was observed as an early-immediate response of E-cadherin adhesion formation [42,48–50], and PI3K seemed to play a critical role in the E-cadherin-mediated activation of Rac [51]. Furthermore, inhibition of PIK3 activity prevented the E-cadherin-mediated activation of Rac [49]. As mentioned earlier, E-cadherin recruits and activates PI3K at sites of cell–cell contact [42–44]. Guanine nucleotide exchange factors (GEFs), which activate Rho GTPases by promoting the exchange of GDP for GTP, were found to recognize activated PI3K through their pleckstrin homology (PH) domains [52]. These results suggest that PI3K may be an upstream activator of Rac in E-cadherin-mediated cell signalling.

**Fig. 3:**
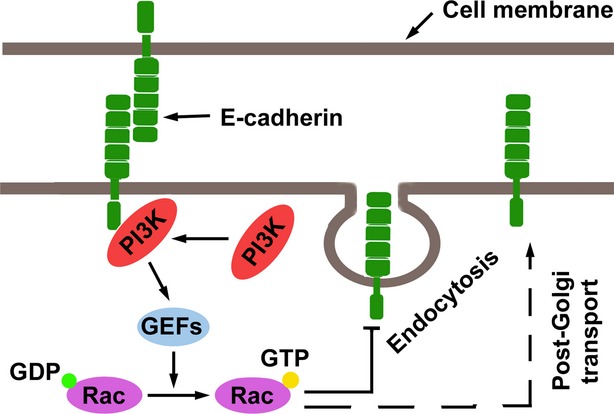
Effects of E-cadherin on the GTPase signalling. E-cadherin-mediated cell–cell contacts activate Rac through activating PI3K, and the activated Rac prevents endocytosis of E-cadherin and promotes the post-Golgi transport of E-cadherin.

In addition to Rac, E-cadherin-mediated cell–cell contact also activated Cdc42 and Rho [53,54]. Interestingly, activation of Rac and Cdc42 appears critical for inducing the formation of AJs in cooperation with E-cadherin [42,55,56]. E-cadherin is known to undergo endocytosis upon disruption of AJs. Notably, the activation of Rac and Cdc42 GTPases was demonstrated to inhibit the endocytosis of trans-interacting E-cadherin in epithelial cells [57,58]. Rac and Cdc42 were also necessary to correctly regulate the post-Golgi transport of E-cadherin and the maintenance of cell polarity [59]. Recently, Cdc42 was reported to promote ubiquitination and lysosomal degradation of E-cadherin through the up-regulation of EGFR signalling and subsequent activation of Rac in breast cancer cells [60]. Furthermore, the activation of RhoA or RhoC inhibited the expression of E-cadherin in metastatic prostate cancer cells [61]. Collectively, these data suggest that the complex bilateral regulation of E-cadherin and Rho GTPases may be affected by a number of factors (Fig. 3).

### Effects of E-cadherin on the NF-κB signalling pathway

**Fig. 4:**
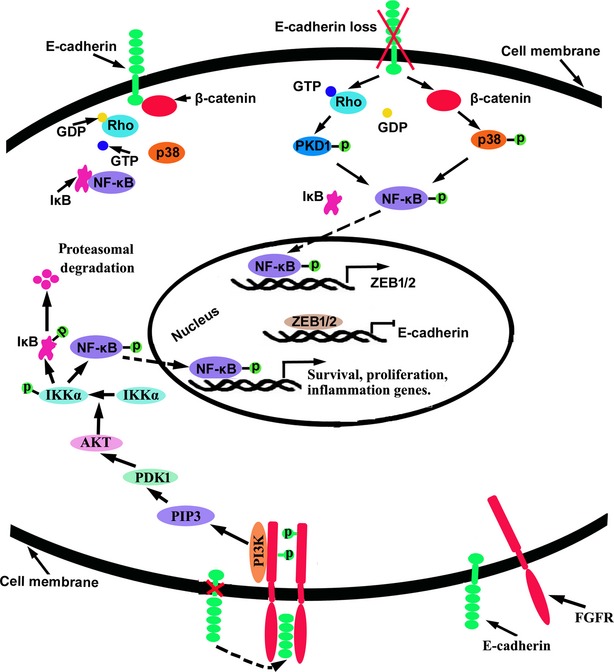
Effects of E-cadherin on the NF-κB signalling. The loss of E-cadherin elevates NF-κB signalling through activating β-catenin and Rho GTPase.

In most cases, E-cadherin negatively regulates NF-κB activation. Studies showed that the loss of E-cadherin and the loss of cadherin-mediated cell–cell contacts activated NF-κB signalling, while the overexpression of E-cadherin suppressed its activity [62,63]. In melanoma cells, the loss of E-cadherin promoted the activation of cytoplasmic β-catenin, which subsequently induced P38-mediated NF-κB activation [63]. In epithelial cells, the dissociation of cell–cell contacts led to the activation of RhoA, which subsequently activated protein kinase D1 (PKD1), a downstream target of RhoA, ultimately inducing the activation of NF-κB [62]. Furthermore, it was demonstrated that restoring E-cadherin expression in colon cancer cells decreased the expression of mesenchymal genes, such as those encoding fibronectin and LEF1, through the inhibition of β-catenin and NF-κB signalling [19] (Fig. 4). However, E-cadherin activity also leads to the expression of tumour suppressors through the up-regulation of NF-κB activity. For example, the decrease of E-cadherin as a result of the activation of MAPK signalling resulted in the down-regulation of neutrophil gelatinase-associated lipocalin (NGAL), a tumour metastasis suppressor that blocks invasion and angiogenesis, through inhibition of NF-κB activation in pancreatic cancer cells. Overexpressing E-cadherin subsequently elevated NF-κB activity and restored the expression of NGAL [64]. Notably, activated NF-κB inhibited the expression of E-cadherin by elevating transcriptional repressors of E-cadherin, such as Snail and ZEB1/2, in multiple cancer types [65–68]. These data suggest the existence of feedback regulation between E-cadherin and NF-κB signalling.

### Mediation of cross-talk between signalling pathways by E-cadherin and p120

**Fig. 5:**
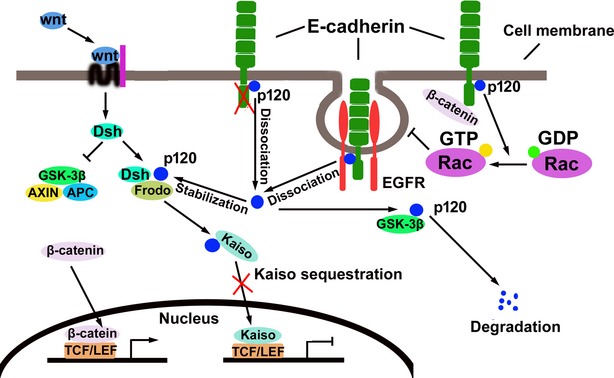
Mediation of cross-talk between signalling pathways by E-cadherin and p120. p120 binds to the cytoplasmic domain of E-cadherin and helps to maintain cell–cell contact by preventing the endocytosis of E-cadherin and stabilizing the cadherin–catenin complex. The loss of E-cadherin and the activation of Wnt signalling stabilize p120 and inhibit Kaiso translocation to the nucleus by forming a p120–Kaiso complex in cytoplasm.

p120 catenin (p120ctn or p120), a member of the catenin family, binds to the cytoplasmic region of E-cadherin and helps to maintain cell–cell contact by preventing the endocytosis of E-cadherin and stabilizing the cadherin–catenin complex [69]. p120 has been found to play an important role in the cross-talk between members of E-cadherin-mediated cell signalling. On the one hand, certain signalling pathways have been shown to regulate the expression and function of E-cadherin through p120. For example, EGF promoted the endocytosis of E-cadherin through regulating p120 activity and, thus, decreasing E-cadherin levels in the cell membrane [34]. Additionally, Wnt signalling pathway activation resulted in Frodo-mediated stabilization of p120 [70]. These results suggest that diverse signalling pathways might affect E-cadherin-mediated signalling through regulating the activity of p120. On the other hand, E-cadherin also affected the distribution and function of p120 [4,71]; thus, E-cadherin itself may regulate other signalling activity through p120. Indeed, p120 has been documented to regulate both GTPase and β-catenin activity (Fig. 5).

The role of p120 in the regulation of Rho GTPase was extensively reviewed previously [72,73]. In summary, p120 was found to directly interact with and regulate Rho GTPase and indirectly modulate Rho activity through interacting with and regulating Rho GEFs. Additionally, p120 was able to promote or suppress the activation of Rho GTPases in different situations. For example, p120 dominantly inhibited Rho activity, but consistently activated Rac and Cdc42 [73]. Furthermore, GTPase regulation could occur either at the site of E-cadherin-mediated cell–cell contacts or in the cytoplasm. When associated with E-cadherin, p120 modulated local GTPases and affected cytoskeletal structures; once dissociated from E-cadherin, p120 could diffuse into the cytoplasm and activate GTPases, thereby affecting the expression of genes involved in a variety of cellular processes, including cell-cycle regulation [74].

Alternatively, liberated p120 could enter the nucleus to regulate gene transcription directly. Like β-catenin, p120 has an Arm-repeat domain, and proteins with this domain may have dual localization at cell–cell junctions and in the nucleus [75–77]. In the nucleus, p120 was reported to interact with the zinc finger transcriptional repressor Glis2 and induce its C-terminal cleavage, although the mechanism of action for this process is unknown [81]. Nuclear p120 was also shown to interact with the BTB/POZ transcriptional repressor Kaiso, inhibiting Kaiso transcriptional activity [76–78]. Kaiso is an inhibitor of the Wnt signalling pathway, directly inhibiting the transcription of Wnt11 [78] and the expression of Wnt signalling targets, such as c-Myc, cyclin D1 and matrilysin (MMP-7), through competitive binding of TCF/LEF with β-catenin [79,80]. The inhibitory role of Kaiso on Wnt signalling and Wnt signalling targets can be attenuated by p120, suggesting that p120 may play a positive role in activation of the Wnt signalling pathway [78–80]. Interestingly, it was demonstrated that Wnt signalling activation stabilized p120, which in turn promoted Kaiso sequestration or removal from the nucleus and elevated Wnt signalling [70]. These data therefore suggest the existence of a possible positive feedback circuit between p120 and Wnt signalling activity.

## Conclusion and perspectives

Over the past decade, E-cadherin has been reported to function as a gene transcriptional regulator, but further studies are needed to more clearly define its likely numerous modes of action in this process. The well-known associations of E-cadherin-mediated AJs with multiple signalling pathways leave little room for doubt that altering E-cadherin would also affect gene transcription through impacting cell signalling. This hypothesis provides a model that signals originating from E-cadherin relay ultimately to the nucleus by molecules that play a central role in the associated signalling pathways. Given the complexity of interaction between E-cadherin-mediated AJs and cell signalling and the existence of cross-talk among different pathways, however, the discrete contribution of each pathway to E-cadherin-mediated gene transcriptional modulation is currently difficult to ascertain. The combined knockdown of E-cadherin and relevant pathway-related molecules may be a useful strategy for tackling this issue.

Nuclear translocation of E-cadherin has also been observed in numerous cancer cell lines and tissues [82–86], which raises the following questions: What is the function of nuclear E-cadherin, and does it directly regulate gene transcription? Ferber *et al*. reported that a cleaved cytoplasmic domain of E-cadherin could enter the nucleus, form a complex with DNA *via* p120 and regulate gene transcription [87]. These data imply the possibility that E-cadherin itself may function in the nucleus as a novel transcriptional regulator, which is definitely an interesting topic and deserving of further systematic study.
